# Quantifying unobserved protein-coding variants in human populations provides a roadmap for large-scale sequencing projects

**DOI:** 10.1038/ncomms13293

**Published:** 2016-10-31

**Authors:** James Zou, Gregory Valiant, Paul Valiant, Konrad Karczewski, Siu On Chan, Kaitlin Samocha, Monkol Lek, Shamil Sunyaev, Mark Daly, Daniel G. MacArthur

**Affiliations:** 1 Department of Biomedical Data Science, Stanford University, Palo Alto, California 94305, USA; 2 Computer Science Department, Stanford University, Palo Alto, California 94305, USA; 3 Computer Science Department, Brown University, Providence, Rhode Island 02912, USA; 4 Analytic and Translational Genetics Unit, Massachusetts General Hospital, Boston, Massachusetts 02114, USA; 5 Broad Institute or MIT and Harvard, Cambridge, Massachusetts 02142, USA; 6 Computer Science and Engineering, Chinese University of Hong Kong, Hong Kong, China; 7 Division of Genetics, Brigham and Women's Hospital, Harvard Medical School, Boston, Massachusetts 02115, USA; 8 Department of Medicine, Harvard Medical School, Boston, Massachusetts 02115, USA

## Abstract

As new proposals aim to sequence ever larger collection of humans, it is critical to have a quantitative framework to evaluate the statistical power of these projects. We developed a new algorithm, UnseenEst, and applied it to the exomes of 60,706 individuals to estimate the frequency distribution of all protein-coding variants, including rare variants that have not been observed yet in the current cohorts. Our results quantified the number of new variants that we expect to identify as sequencing cohorts reach hundreds of thousands of individuals. With 500K individuals, we find that we expect to capture 7.5% of all possible loss-of-function variants and 12% of all possible missense variants. We also estimate that 2,900 genes have loss-of-function frequency of <0.00001 in healthy humans, consistent with very strong intolerance to gene inactivation.

## Abstract (web summary)

Accurate estimations of the frequency distribution of rare variants are needed to quantify the discovery power and guide large-scale human sequencing projects. This study describes an algorithm called UnseenEst to estimate the distribution of genetic variations using tens of thousands of exomes.

## Graphical abstract

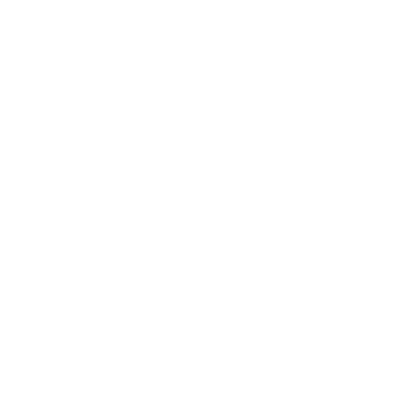

Recent efforts aggregating the genomes and exomes of tens of thousands of individuals have provided unprecedented insights into the landscape of rare human genetic variation^1^,^2^ and generated critical resources for clinical and population genetics. The recently announced U.S. Precision Medicine Initiative raises the prospect of growing these databases to encompass hundreds of thousands of human genomes. In the context of these ambitious efforts, it is important to quantify the power of large sequencing projects to discover rare functional genetic variants^3^. In particular, we need to understand, as we sequence ever larger cohorts of individuals, how many new variants we can expect to identify and their expected allele frequencies. Accurate estimates of these quantities will enable better study design and quantitative evaluation of the potential and limitations of these datasets for precision medicine.

Predicting the number of new variants, we expect to identify in larger cohorts requires accurate estimates of allele frequencies of all the genetic variation in the human population, including the rare variants that have not been observed in the current sequencing cohorts^4^,^5^,^6^. As common variants have already been saturated in the current cohorts, the population frequencies of the unobserved rare variants determine the discovery rate of new variants as the cohort sizes increase. These rare variants also include many variants with large effect sizes, which are targets of disease-sequencing studies^7^. The frequency distribution of rare variants thus reflects a confluence of several important processes including the demographic history of the population^8^,^9^,^10^ and the forces of selection acting on these variants^11^,^12^.

Estimating the frequency distribution of genetic variation is closely related to the classic statistics problem of estimating the number of unseen animal species from capture experiments^13^,^14^. Leveraging this connection, previous methods used Bayesian and jackknife approaches to estimate the discovery rate of new variants^4^,^5^,^15^. The Bayesian methods estimate the discovery rate by using specific prior distributions on the variant frequencies, which enable tractable computation^4^. The parametric forms of the prior typically correspond to the assumption that the variants are selectively neutral, and this assumption could lead to biased predictions especially on missense and truncation variants. Unlike the Bayesian approach, which models the variant frequency distribution, the jackknife approach directly models the variant discovery rate using a parametric form derived from consistency requirements (see ‘Methods' section) that does not need to assume neutrality. The jackknife is validated to produce accurate 20-fold extrapolation on small cohorts, such as the individuals from the 1,000 Genome populations^16^.

We developed a new method, UnseenEst, to estimate the frequency distribution of all genetic variants in the population based on the observed site-frequency spectrum (SFS) of the current cohort. UnseenEst does not assume a prior distribution of variant frequencies and is applicable across different demographic and selection models. We validated and applied UnseenEst on a significantly larger dataset than was previously explored—the exome sequencing data of 60,706 individuals assembled by the Exome Aggregation Consortium (ExAC)^17^. For several classes of variants, we systematically estimated how many distinct new variants would be discovered as we further scale up the sequencing effort. We also provide estimates for more complex statistics of biomedical interest, such as the expected number of genes harbouring multiple loss-of-function (LoF) variants in expanded cohorts.

## Results

### Quantifying unobserved protein-coding variants

UnseenEst uses a linear programme to identify the variant frequency distribution whose expected SFS most closely matches the empirically observed SFS. Recent works have successfully explored using different linear programs for related tasks such as estimating entropy^18^ and estimating the upper and lower bounds on the number of distinct genetic features^5^. In comparison, UnseenEst directly aims to estimate the frequency distribution itself. Because the objectives functions are different, the previous linear programs differ from UnseenEst in their formulations and modelling assumptions—for example, the infinite genome assumption in Gravel^5^ is not needed for UnseenEst. More detailed discussions of these methods are included in the ‘Methods' section and the Supplementary Information. Our empirical and mathematical analysis shows that UnseenEst provides accurate extrapolation of the SFS from current data to cohort sizes more than an order of magnitude larger (Supplementary Notes 1–6).

Protein-coding variants represent the most readily interpretable and medically relevant slice of human genetic variation, and have been assessed in large sample sizes through the widespread application of exome sequencing approaches^2^. We leveraged data from the ExAC to estimate the discovery rates of different classes of protein-coding variants in larger cohorts. We validated UnseenEst by training it on random 10% of the alleles in ExAC and then used the estimated frequency distribution to predict the number of distinct variants that we can identify in the entire ExAC cohort. For every variant type (Supplementary Fig. 1) and every population (Supplementary Fig. 2), UnseenEst accurately predicted the number of unique variants that were identified in the entire ExAC cohort as well as the empirical SFS of ExAC (Supplementary Table 1). UnseenEst does not make parametric assumptions about the shape of the variant frequency distributions. This important feature enables it to accurately estimate the frequency distribution of variants that have evolved under different selection pressures (for example, synonymous and missense variants) as well as under different demographic histories. Commonly used methods for predicting variant discovery rates, such as the jackknife estimator^5^, assume that the discovery rate has a particular parametric form, which can lead to biases when there is a model mismatch. On the same validation experiments using random 10% of ExAC alleles, jackknife consistently underestimated the true number of distinct variants in all populations (Supplementary Fig. 3).

From the full ExAC dataset, we generated a cohort of 33,778 healthy individuals that matched the ancestral population breakdown of the 2010 U.S. Census (Supplementary Table 2). We trained UnseenEst on this U.S. Census-matched cohort and predicted the frequency distributions of variants in the entire population (Supplementary Fig. 4). In particular, we estimated the number of distinct variants we expect to identify in cohorts of up to 500K individuals. These results provide a quantitative framework to evaluate the power and limitations of precision medicine initiatives in discovering rare coding variants.

**Figure 1:**
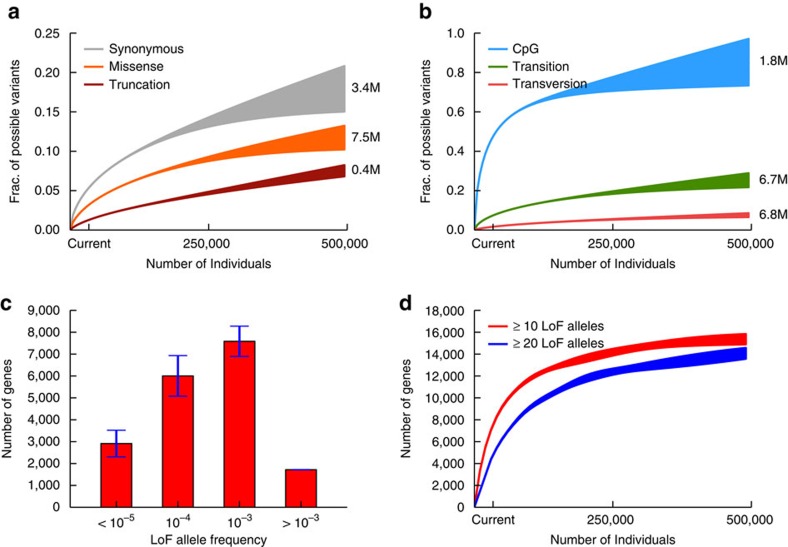
Predictions for the number of unique variants in 500K individuals. We trained UnseenEst on the U.S. Census-matched ExAC cohort (‘current') and predicted the number of unique variants we expect to find in up to 500K individuals. The number of unique variants in the cohort were estimated for synonymous, missense and LoF variants in (**a**), and for CpGs, transitions and transversions in (**b**). The shaded regions correspond to one standard deviation around the estimates. (**c**) A gene is classified as LoF on a given allele if that allele contains at least one variant that introduces a stop codon, disrupts a splice donor/receptor site or disrupts the reading frame. Genes are partitioned into bins based on their LoF allele frequencies: <10^−5^, 10^−5^–10^−4^, 10^−4^–10^−3^ and >10^−3^. The *y* axis indicates the number of genes with LoF allele frequency belonging to each bin. Error bars correspond to one standard deviation. (**d**) Estimated number of genes with at least 10 and 20 LoF alleles.

We categorized the variants by their predicted functional consequence—synonymous, missense and LoF, which is defined as point substitutions that introduce stop codons or disrupt splice donor/acceptor sites (Fig. 1a; Supplementary Table 3). The discovery rate of LoF variants is the lowest, reflecting the fact that LoFs are likely to be deleterious and hence tend to occur comparatively rarely in the healthy population. With 500K individuals, we expect to identify 400K distinct LoF variants or 7.5% of all possible LoF point mutations in the human exome. In the same cohort, we expect to identify 3.4 million synonymous and 7.5 million missense variants, corresponding to 18 and 12% of possible synonymous and missense variants, respectively. These estimates indicate that the discovery rates of rare LoF, missense and synonymous variants are far from saturation, even with 500K individuals. We note that slightly higher numbers of distinct synonymous and missense variants (Supplementary Fig. 5) would be discovered if the 500K individuals were instead sampled from the same ancestral composition as the current ExAC cohort, which contains higher fractions of South and East Asian individuals than the United States, confirming that the overall discovery rate of rare variants can be boosted by optimizing the population composition of the sequencing cohort^19^.

We additionally classified the variants by their biochemical properties (Fig. 1b). With the 34K individuals of the current cohort, we can already identify close to 50% of all possible variants at CpG sites (the most highly mutable substitution class), and the discovery rate for this class of variant quickly saturates as cohorts grow larger. Transversions, in contrast, are discovered much more slowly—attaining 7.6% of all possible transversions with 500K individuals—which is consistent with their much lower mutation rate. We further applied UnseenEst to quantify the number of distinct missense variants we expect to discover in specific gene families of interest, for example genes near genome-wide association study (GWAS) hits and known drug target genes (Supplementary Fig. 6). Missense mutations in drug target genes are particularly suppressed, suggesting that these genes are more likely to be essential to humans.

LoF variants likely disrupt the normal function of genes and by studying individuals carrying such variants, we can quantify the phenotypic consequence of disrupting particular genes. Therefore, a catalogue of the number of human alleles harbouring candidate LoF variants for each gene is an important resource for drug development and disease diagnosis. We applied UnseenEst to estimate the LoF frequency of genes in the U.S. population (Fig. 1c; Supplementary Fig. 7). About 2,900 genes have LoF allele frequency lower than 10^−5^, consistent with strong intolerance to inactivation, whereas 1,700 genes are expected to harbour LoF variants in at least 0.1% of the population. With 250K individuals, we expect to identify 14K genes that harbour LoFs in at least 10 individuals, substantially expanding the current catalogue of 10K such genes in ExAC (Fig. 1d; Supplementary Fig. 8). We estimate that the discovery rate of these genes with multiple LoF occurrences will saturate around 16 K, providing an upper bound on the number of genes that can tolerate LoF variants on one allele.

## Discussion

We describe a framework for estimating the power of sequencing cohorts to discover protein-coding variants. We apply it to the largest available collection of sequenced individuals to estimate the discovery power of much larger cohorts such as the ones proposed by the Precision Medicine Initiative. While our predictions here assumed that the samples are representative of the U.S. demography, UnseenEst can be directly applied to estimate the discovery rate of cohorts with different ancestral composition.

A key assumption of UnseenEst and of all the methods for estimating unseen variants is that the alleles in the cohort are random samples of alleles from the population^5^. Here, we used weighted sampling of individuals in the ExAC data to match the U.S. census demography. Because the census relies on self-reported ethnicity, the match based on genetic ancestry may not be perfect. Moreover, the actual cohort assembled for the Precision Medicine Initiative and other large-scale projects will likely deviate from the U.S. census demography due to study designs and practical constraints. The discovery rates estimated here can be viewed as a baseline. Adjustment will need to be made for each specific cohort, for example by using a different weighted sampling of the ExAC data. Using training data from the current ExAC cohort, the UnseenEst predicted discovery rates have wide confidence intervals for study sizes >500,000 participants. This uncertainty stems from the uncertainty in the frequency distribution of very rare variants, most of which have not been observed in ExAC. Reducing this uncertainty without introducing too much bias would be a useful advance. This paper focuses on the discovery rate of single-nucleotide variants. Another important question of future work is to develop methods to estimate the discovery power of more complex types of genetic variation such as insertion/deletions.

Naturally occurring LoF variants provide a powerful model for understanding the impact of gene inactivation on human phenotype, as illustrated by the development of PCSK9 inhibitors (guided by the discovery of LoF variants in this gene that reduce LDL cholesterol)^20^. For researchers interested in leveraging ‘human knockout' data in drug development and disease diagnosis, the UnseenEst predictions rigorously quantify the statistical powers of the future cohort-sequencing projects. This can inform both cohort designs as well as downstream analysis. For example, UnseenEst predicts that in a cohort of 250 K individuals, we would find around 14K genes with at least 10 individuals carrying protein-truncating variants (Fig. 1d), who could then be followed up to understand the impact of heterozygous LoF of that gene on human phenotypes. Overall, our results show that sequencing a cohort of 500K randomly selected U.S. individuals would provide access to over 12% of all possible missense variants and 7.5% of all possible LoF variants, thereby permitting exploration of a substantial fraction of human biological diversity.

## Methods

### UnseenEst algorithm

UnseenEst uses the empirical SFS of a given class of variants in a cohort to estimate its frequency distribution in the population. The inputs into the algorithm are the number of alleles in the sample cohort, *k*, and the SFS, which is a set of counts, {*F*_*i*_}, where *F*_*i*_ is the number of variants that are observed in exactly *i* out of the *k* alleles. A key challenge for the method is to accurately estimate the frequency distribution of variants that have empirical count of 0 (that is, they are not observed) in the cohort but are likely to have some small, non-zero frequency in the population.

More concretely, let *X* denote a discrete set of frequencies *x* in [0, 1] and let *h*(*x*) denote the fraction of all the variants with frequency *x*. UnseenEst estimates *h*(*x*) by finding the set of *h*(*x*) that jointly minimizes the value of the expression:


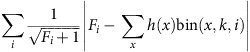


where bin(*x, k, i*) is the binomial probability of observing *i* heads in *k* independent flips of a coin with bias *x.* The intuition for minimizing this objective is as follows: 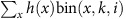 is the number of variants that we expect to find in *i* out of *k* alleles in the cohort and *F*_*i*_is the empirical number of variants observed in *i* alleles. If *h*(*x*) is the true frequency distribution, then the expectation should be close to the empirical, which is why we want to find *h*(*x*) that minimizes the objective above. Given an estimate of the frequency distribution *h*(*x*), the expected number of unique variants in *N* alleles can be calculated by the formula 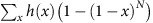.

The standard 3rd order jackknife estimator does not estimate the frequency distribution *h*(*x*). Instead, it directly estimates that the number of unique variants in *N* alleles is *g*_1_(*N*, *k*)*F*_1_*+g*_2_(*N*, *k*)*F*_2_*+g*_3_(*N*, *k*)*F*_3_, where *F*_1_, *F*_2_, *F*_3_ are the number of variants observed once, twice and three times in *k* alleles, and *g*_1_, *g*_2_, *g*_3_ are specific functions of *N* and *k* derived from self-consistency requirements^5^. Note that the confidence intervals of the jackknife in Supplementary Fig. 3 are very narrow because there is relatively little variation in the counts *F*_1_, *F*_2_, *F*_3_,when the sample size is large.

To calculate the gene-level frequency distribution of LoF variants, we let *F*_*i*_ be the number of genes that are observed to have at least one LoF variant in *i* out of *k* alleles. Then the *h*(*x*) produced by UnseenEst can be interpreted as the number of genes with at least one LoF variant in *x* fraction of the population. Supplementary Information contains detailed discussion of the UnseenEst algorithm, analysis of its theoretical properties and relations to other methods.

### Datasets

We used the exome sequencing data from the ExAC^17^. This dataset consists of high-quality sequencing of the protein-coding regions in the genome (exomes) from 60,706 healthy individuals. Consistent with the ExAC analysis, we considered only regions of the exome with sufficient sequencing depth: each nucleotide must be covered by at least 10 reads in at least 80% of all ExAC individuals. Variant annotation was performed using the Variant Effect Predictor^21^ (VEP) v81 on Gencode v19 and genome build GRch37. We define LoF variants to be single-nucleotide substitutions that introduce a stop codon in the reading frame or disrupts a splice donor or receptor site. LoF annotation was performed using LOFTEE (version 0.2) plugin to VEP.

### Validation

To validate UnseenEst, we randomly partitioned all the ExAC alleles into 10 groups. For each class of variant (synonymous, missense, LoF, CpG), we trained UnseenEst on the SFS of one partition (that is, 10% of the alleles) and used the model to predict the allele frequency distribution as well as the discovery rates of the entire ExAC cohort. The confidence intervals correspond to the standard deviation of the predictions across different partitions.

### Data availability

The ExAC data used in the paper is publicly available and can be downloaded at http://exac.broadinstitute.org/. The UnseenEst software is implemented in Python and can be downloaded from https://github.com/jameszou/unseenest.

## Additional information

**How to cite this article:** Zou, J. *et al*. Quantifying unobserved protein-coding variants in human populations provides a roadmap for large-scale sequencing projects. *Nat. Commun.* **7,** 13293 doi: 10.1038/ncomms13293 (2016).

**Publisher's note:** Springer Nature remains neutral with regard to jurisdictional claims in published maps and institutional affiliations.

## Supplementary Material

Supplementary Information Supplementary Figures 1-8, Supplementary Tables 1-3, Supplementary Notes 1-6 and Supplementary References

## References

[b1] Auton A. . A global reference for human genetic variation. Nature 526, 68–74 (2015). 26432245 10.1038/nature15393 PMC4750478

[b2] Macarthur D. G. . A systematic survey of loss-of-function variants in human protein-coding genes. Science 335, 823–829 (2012). 22344438 10.1126/science.1215040 PMC3299548

[b3] Collins F. S. & Varmus H. A new initiative on precision medicine. N. Engl. J. Med. 372, 793–795 (2015). 25635347 10.1056/NEJMp1500523 PMC5101938

[b4] Ionita-Laza I., Lange C. M. & Laird N. Estimating the number of unseen variants in the human genome. Proc. Natl Acad. Sci. USA 106, 5008–5013 (2009). 19276111 10.1073/pnas.0807815106 PMC2664058

[b5] Gravel S. Predicting discovery rates of genomic features. Genetics 197, 601–610 (2014). 24637199 10.1534/genetics.114.162149 PMC4063918

[b6] Henn B. M., Botigué L. R., Bustamante C. D., Clark A. G. & Gravel S. Estimating the mutation load in human genomes. Nat. Rev. Genet. 16, 333–343 (2015). 25963372 10.1038/nrg3931 PMC4959039

[b7] Zuk O. . Searching for missing heritability: designing rare variant association studies. Proc. Natl Acad. Sci. USA 111, E455–E464 (2014). 24443550 10.1073/pnas.1322563111 PMC3910587

[b8] Luikart G., Allendorf F., Cornuet J.-M. & Sherwin W. Distortion of allele frequency distributions provides a test for recent population bottlenecks. J. Hered. 89, 238–247 (1998). 9656466 10.1093/jhered/89.3.238

[b9] Gutenkunst R. N., Hernandez R. D., Williamson S. H. & Bustamante C. D. Inferring the joint demographic history of multiple populations from multidimensional SNP frequency data. PLoS Genet. 5, e1000695 (2009). 19851460 10.1371/journal.pgen.1000695 PMC2760211

[b10] Durrett R. & Limic V. On the quantity and quality of single nucleotide polymorphisms in the human genome. Stoch. Process. Appl. 93, 1–24 (2001).

[b11] Akey J. M., Zhang G., Zhang K., Jin L. & Shriver M. D. Interrogating a high-density SNP map for signatures of natural selection. Genome Res. 12, 1805–1814 (2002). 12466284 10.1101/gr.631202 PMC187574

[b12] Park J.-H. . Distribution of allele frequencies and effect sizes and their interrelationships for common genetic susceptibility variants. Proc. Natl Acad. Sci. USA 108, 18026–18031 (2011). 22003128 10.1073/pnas.1114759108 PMC3207674

[b13] Efron B. & Thisted R. Estimating the number of unseen species: how many words did Shakespeare know? Biometrika 63, 435–447 (1976).

[b14] Burnham K. P. & Overton W. S. Estimation of the size of a closed population when capture probabilities vary among animals. Biometrika 65, 625–633 (1978).

[b15] Gravel S. . Demographic history and rare allele sharing among human populations. Proc. Natl Acad. Sci. USA 108, 11983–11988 (2011). 21730125 10.1073/pnas.1019276108 PMC3142009

[b16] Abecasis G. R. . An integrated map of genetic variation from 1,092 human genomes. Nature 491, 56–65 (2012). 23128226 10.1038/nature11632 PMC3498066

[b17] Lek M. . Analysis of protein-coding genetic variation in 60,706 humans. Nature 536, 285–291 (2016). 27535533 10.1038/nature19057 PMC5018207

[b18] Valiant P. & Valiant G. Estimating the unseen: improved estimators for entropy and other properties. In *Advances in Neural Information Processing Systems 26* (NIPS, 2013).

[b19] Ionita-Laza I. & Laird N. M. On the optimal design of genetic variant discovery studies. Stat. Appl. Genet. Mol. Biol. 9, Article33 (2010). 20812911 10.2202/1544-6115.1581 PMC2942028

[b20] Cohen J. C. . Multiple rare alleles contribute to low plasma levels of HDL cholesterol. Science 305, 869–872 (2004). 15297675 10.1126/science.1099870

[b21] McLaren W. . The Ensembl variant effect predictor. Genome Biol. 17, 122 (2016). 27268795 10.1186/s13059-016-0974-4 PMC4893825

